# Innovative Strategies to Reach Diverse Elders: Using Age-Tastic to Improve Health and Well-Being

**DOI:** 10.1093/geroni/igaa057.1206

**Published:** 2020-12-16

**Authors:** Jacquelin Berman, Manoj Pardasani, Madison Gates, Mebane Powell

**Affiliations:** 1 NYC Department for the Aging, NYC, New York, United States; 2 Hunter school of social work, New York city, New York, United States; 3 NYC Department for the Aging, New York city, New York, United States

## Abstract

This session will present the findings of a randomized control trial evaluating the impact Age-Tastic has on behavioral change. Age-Tastic is an eight-week intervention that uses game play, group facilitated discussion, and at home activities to promote positive behavioral change. There are five behavioral change domains: emotional well-being, nutrition, financial exploitation, falls prevention, and health literacy. A randomized control trial was conducted in New York City senior centers and include a diverse group of older adults. There were 98 older adults assigned to an experimental or control group. Participants in both groups completed a baseline survey, which was repeated at the end of the intervention and at week sixteen. The experimental (n = 64) and control (n = 34) groups did not significantly differ at baseline in regard to behaviors; however, upon completion of the intervention, the experimental group had significantly changed health behaviors (p < .05) compared to the control group. The behavior change reported by the experimental group was maintained at week sixteen (p < .001) with a medium effect size (ε2 = .17). In addition the experimental group also reported significant improvements at weeks eight (p < .001) and sixteen (p < .001) with large effect sizes (r2 = .62 and .52, respectively). This study found that Age-Tastic is an effective intervention for engaging older adults holistically about their health and wellness and for promoting positive behavioral change among diverse racial and ethnic populations.

